# Comparison of Design, Cyclic Fatigue Resistance, and Metallurgical Properties of Original, Replica‐Like, and Counterfeit Nickel‐Titanium Files

**DOI:** 10.1002/jemt.70061

**Published:** 2025-08-12

**Authors:** Mert Unal, Elif Bahar Cakici

**Affiliations:** ^1^ Department of Endodontics, Faculty of Dentistry Ordu University Ordu Turkey

**Keywords:** counterfeit, cyclic fatigue, nickel‐titanium, original, replica‐like

## Abstract

This study aimed to compare the design characteristics, cyclic fatigue resistance, and metallurgical properties of original, replica‐like, and counterfeit nickel‐titanium systems. One hundred Ni‐Ti files were evaluated and categorized into four groups: the original (Of‐Reciproc Blue R25) system, a replica‐like (Rf‐Recip‐One Files Blue R25) system, and two counterfeit (Cf1, Cf2) systems. The design characteristics were assessed based on packaging features, manufacturing defects observed using a stereomicroscope, tip design analyzed via scanning electron microscopy (SEM), and taper and tip diameter measurements conducted with Image J software (National Institutes of Health, Bethesda, MD, USA). Cyclic fatigue testing was performed at body temperature in an artificial canal with a 60° angle of curvature and a 5 mm radius of curvature. Metallurgical properties were examined using differential scanning calorimetry (DSC) and energy‐dispersive spectroscopy (SEM‐EDS). Statistical analysis was conducted using the Kruskal–Wallis test with post hoc Dunn‐Bonferroni tests, with the significance level set at 5%. The Cf systems were distinguished from the Of system by the observation of manufacturing defects under a stereomicroscope. While the Of and Rf systems exhibited passive tip designs, the Cf systems displayed active tip designs. The Rf system showed tip diameter and taper values similar to those of the Of system, whereas the Cf1 system demonstrated lower tip diameter and taper values compared to the Of system. Cyclic fatigue test results revealed no statistically significant difference in fracture times between the systems. DSC analysis indicated that the Of system was in the austenite phase at body temperature, while the other systems were in the martensite phase. SEM‐EDS analysis revealed similar nickel‐titanium compositions across all systems. The Rf system showed similar design, mechanical, and metallurgical properties to the Of system, while Cf systems lacked consistency in standardization and design. The presence of manufacturing defects, along with discrepancies in claimed design specifications such as tip diameter and taper, and the fact that counterfeit systems exist in a different metallurgical phase compared to the original system, may predispose clinicians to potential complications during clinical use.

## Graphical abstract

Evaluation of the design, cyclic fatigue resistance, and metallurgical properties of original, replica‐like, and counterfeit nickel‐titanium endodontic files.

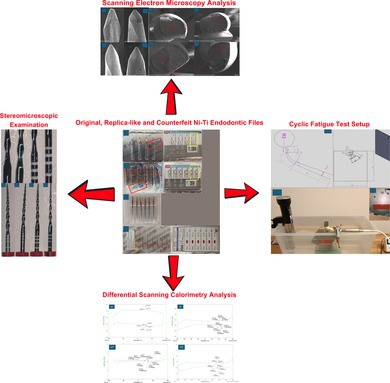

**Summary**

- Counterfeit systems were distinguished from the original system by identifying manufacturing defects under a stereomicroscope.
- No statistically significant difference was found between the systems in terms of resistance to cyclic fatigue.
- While replica‐like system exhibit similar design, mechanical, and metallurgical properties to the original system, counterfeit systems have been found to be inconsistent in terms of design and standardization.

## 1 Introduction

The instruments are critical for cleaning and shaping the canal system (Young et al. 2007). Using nickel‐titanium files has introduced several advantages compared to conventional stainless steel hand files, including faster preparation times, improved cutting efficiency, and enhanced canal centering ability (Cheung and Liu 2009). Despite these advantages, the risk of file fracture remains a complication of root canal treatment (Adorno et al. 2009; Kim et al. 2010). Instrument fracture within the canal can occur due to torsional or cyclic fatigue (Sattapan et al. 2000). A torsional fracture occurs when the file tip becomes locked in the canal while the shaft continues to rotate, resulting in a torque exceeding the metal's elastic limit (Keskin et al. 2017; Yilmaz et al. 2021). In contrast, cyclic fatigue fracture typically arises during the preparation of curved canals, where the instrument is subjected to repeated compressive and tensile forces at the point of maximum curvature (Cheung 2007; Pedullà et al. 2020). Resistance to cyclic fatigue is influenced by several factors, including file flexibility, cross‐sectional design, cutting angle, size, taper, and environmental temperature (de Melo et al. 2002; Fukumori et al. 2018; Grande et al. 2006; Kosti et al. 2011; Plotino et al. 2009a; De Vasconcelos et al. 2016).

Another major disadvantage of Ni‐Ti systems is their cost. Clinicians have used various methods to avoid these costs (Locke et al. 2013). As a result, a number of file systems have been launched in recent years that are similar to products from well‐known brands such as VDW, Dentsply, and MicroMega. These systems are generally classified under two headings. First systems, known as replica‐like files, imitate the features of recognized brands and have similar characteristics to the original, such as number/sequence of instruments, nomenclature, and identification, but do not provide clear reports on manufacturing quality control or international certification (Martins et al. 2020). The second system, known as counterfeit files, is an imitation product manufactured and packaged under the same name as genuine items. Unlike legitimate and legal products, counterfeit files infringe patent rights, falsely claim regulatory approval, and violate the trademark rights of well‐recognized products (Martins et al. 2022; Rodrigues et al. 2018).

This study aimed to guide clinicians in file selection by comparing the design, mechanical, and metallurgical properties of original, replica‐like, and counterfeit file systems. The null hypothesis stated that there would be no significant differences between the original and alternative files in terms of design features, cyclic fatigue resistance, and metallurgical properties.

## 2 Materials and Methods

**FIGURE 1:**
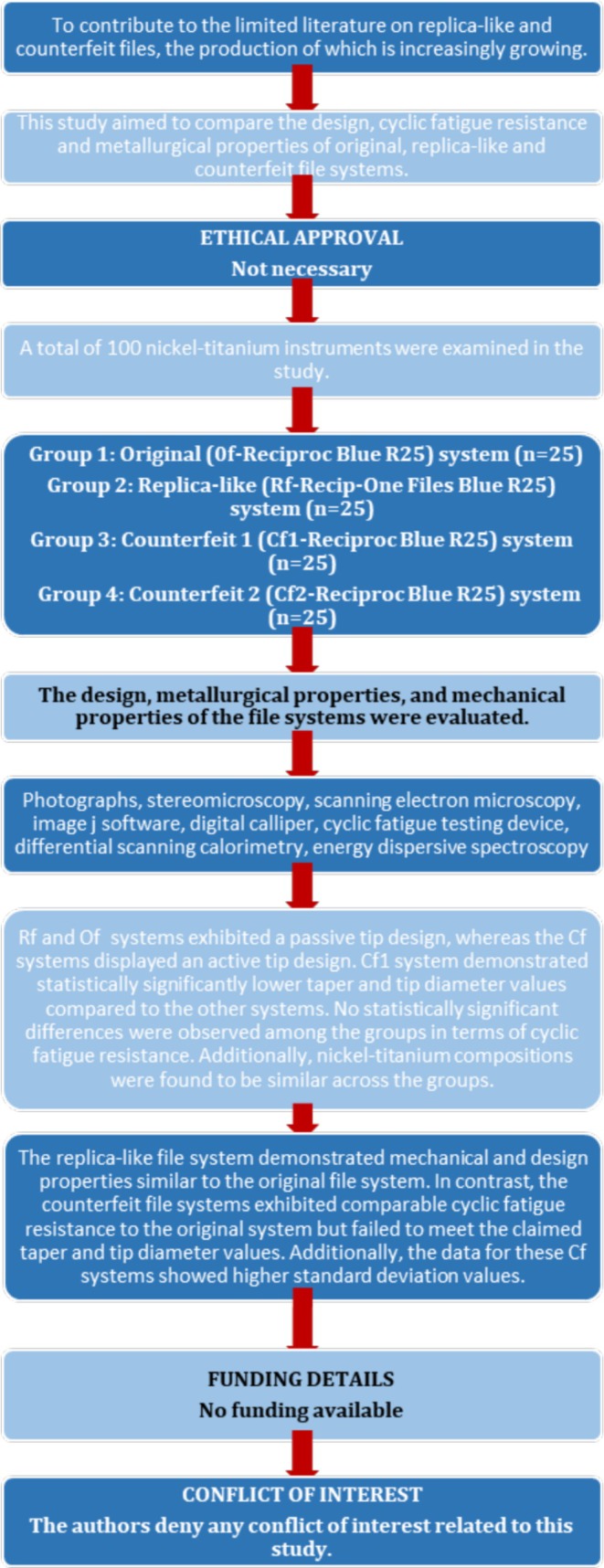
PRILE flowchart.

The manuscript of this laboratory study has been written according to Preferred Reporting Items for Laboratory Studies in Endodontology (PRILE) 2021 guidelines (Figure 1).

One hundred Ni‐Ti files were evaluated for design characteristics, cyclic fatigue resistance, and metallurgical properties. Groups as follows.

Of group: Original systems represented by Reciproc Blue R25 (VDW, Munich, Germany).

Rf group: Replica‐like systems represented by Recip‐One Files Blue R25 (Rogin Dental, Shenzhen, China); and identified by the same numbering or sequence, identical color coding, and similar or identical nomenclature to the original system. Accordingly, replica‐like files meeting these criteria were selected (Martins et al. 2020).

Cf1 group: Counterfeit systems are a complete imitation of the original product and packaging, which is a violation of intellectual property rights (Rodrigues et al. 2018). In accordance with this definition, Reciproc Blue R25 files were obtained from the seller “Shop 1,104,487,846” on the AliExpress platform at approximately one‐sixth the price of the original system (AliExpress 2025a).

Cf2 group: Same as the CF1 group, the files were purchased from a different seller on Aliexpress.com (VDK Dent Store) (AliExpress 2025b).

### 2.1 Design

The packaging and visual characteristics of the file systems from the four groups were examined side by side for similarities. Photographs of the systems were taken using an iPhone 13 Pro Max (Apple Inc., California, USA). Digital images of the file systems were taken using a stereomicroscope (Carl Zeiss Etalon 300, Jena, Germany) at a zoom ratio of 7:1 and within a magnification range of ×10–×25. These images were analyzed for manufacturing defects.

Ten files from each group were randomly selected for examination by SEM (Hitachi SU‐1510, Tokyo, Japan). Tip designs were analyzed at ×200 magnification, while digital images taken at ×50 magnification were evaluated for tip diameter and taper using Image J software (National Institutes of Health, Bethesda, Maryland, USA). To prevent variations in taper and tip diameter measurements due to differences in the positioning and angulation of the files during imaging, all files were fixed in place using an adhesive sample holder. The images were then captured at a consistent magnification and fixed working distance. Tip diameter was measured at the first cutting edge of the file according to the American National Standard/American Dental Association Specification No. 101 (American National Standard 2001). The taper of the file was measured using the formula established by ANSI/ADA Spec 101: Taper = Distance of Diameters/Distance between Diameters. The guideline states that the diameters included in the above equation are D0 and D16 (or alternatively D3 and D16) (American National Standard 2001). The D0 and D1 diameter values of the Reciproc Blue system were specified in the manufacturer's documentation, and these file systems have a variable taper (VDW‐Dental 2025). Consequently, the formula used the D0 and D1 diameters in the calculations.

In addition, for 20 files from each group, the accuracy of the measurement marks at 18 mm and 20 mm was assessed using a digital caliper with an accuracy of 0.01 mm (Absolute AOS series 500; Neuss, Germany). A threshold deviation of 0.1 mm was established, and values above this threshold were considered significant.

### 2.2 Cyclic Fatigue Test

The sample size for the cyclic fatigue test was determined using the G*Power 3.1 software (Macintosh, Düsseldorf, Germany). Based on data from a similar study, an effect size of 0.4, an alpha error probability of 0.05, and a power level of 0.8 indicated that a minimum of 76 samples would be required for four groups (Bürklein et al. 2024). Accordingly, a total of 80 samples, with 20 files in each group, were included in the static cyclic fatigue test.

**FIGURE 2:**
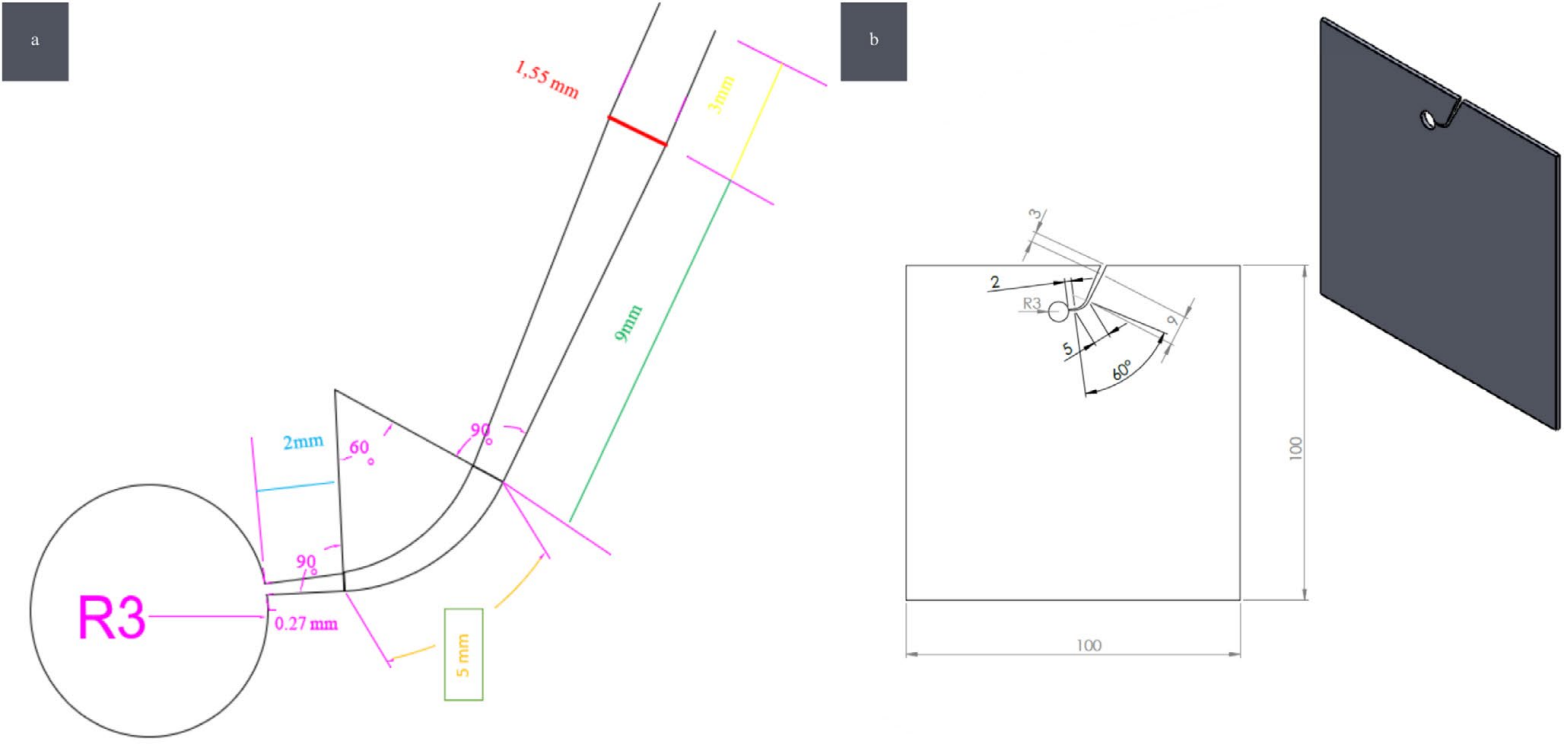
Draft (a) and AutoCAD design of the artificial canal (b).

The artificial canal was designed using AutoCAD software (AutoDesk, San Francisco, USA) and fabricated from stainless steel blocks by CNC machining. The canal design was fabricated in dimensions compatible with the specifications of the instruments under investigation (Plotino et al. 2009b; Plotino et al. 2012; Gambarini et al. 2015; Khalil and Natto 2019) (Figure 2a,b). The artificial canal had an internal diameter of 1.55 mm coronally (D16), tapering at a rate of 0.08 to mimic the shape of the files being tested and narrowing to a diameter of 0.27 mm apically (D0). A tolerance limit of 0.02 mm was included in accordance with ISO standards to ensure that the files could rotate freely within the canal (International Organization for Standardization 2019). The artificial canal was designed with a total length of 19 mm, a 60° angle of curvature, and a 5 mm radius of curvature. The curved segment of the canal was configured to have a length of 5 mm, with the point of maximum curvature located 5 mm from the tip of the instrument. The distance between the point where the curvature ends and the endpoint of the canal was set to 2 mm.

**FIGURE 3:**
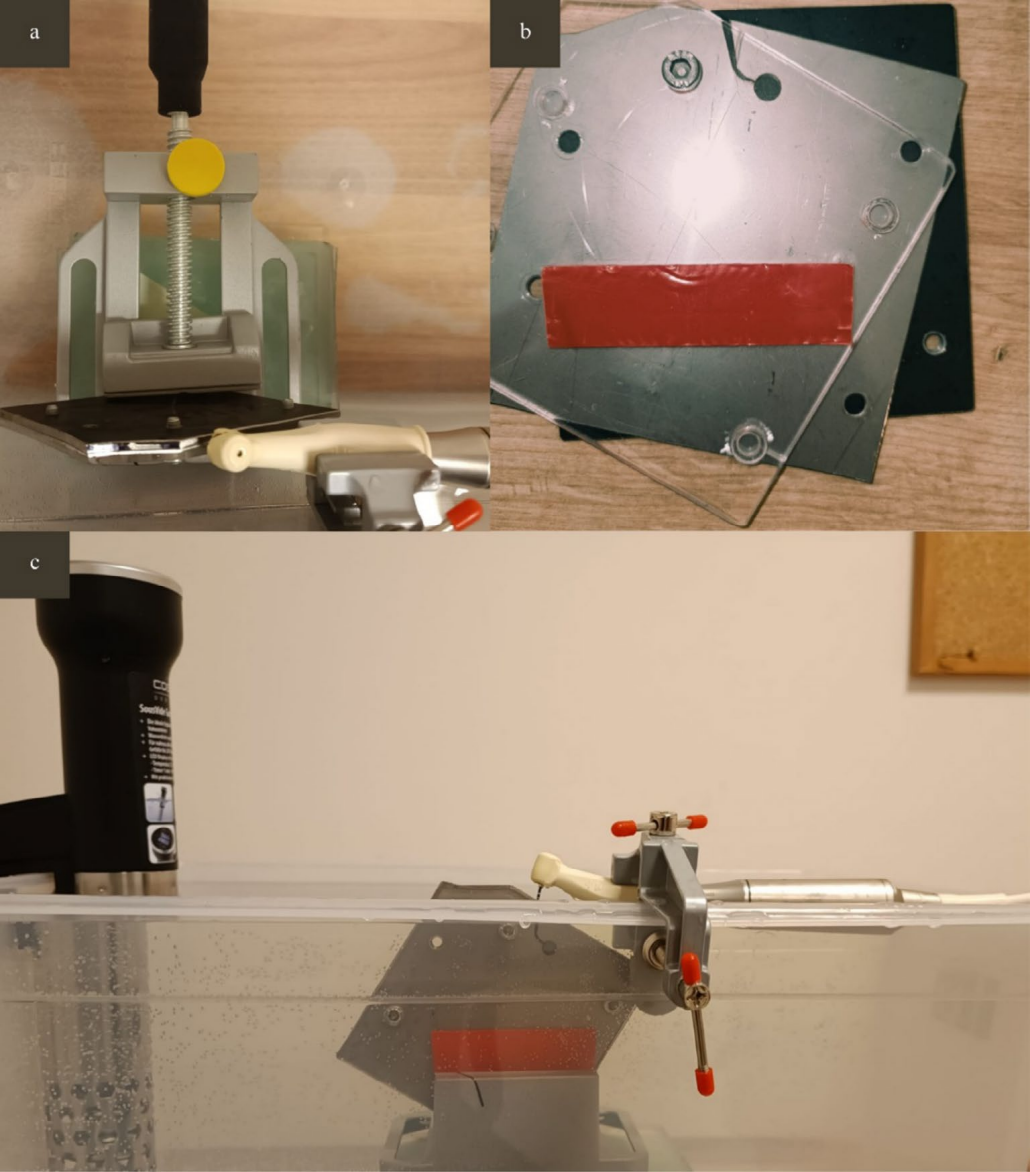
Top view of the cyclic fatigue testing device (a), three‐layered structure of the artificial canal (b), and front view of the cyclic fatigue testing device (c).

The artificial canal was covered with a glass plate to prevent file displacement and damage, while allowing visual observation of the fracture moment (Figure 3). A gastronomic container was filled with 5000 mL of saline solution, and the solution temperature was maintained at 37°C using a sous vide machine (Caso SV 400, Arnsberg, Germany). To ensure standardization of the test setup (Figure 3), the artificial canal was fixed in a transparent gastronomic container using a vise. Another vise was used to align the endodontic handpiece head parallel to the artificial canal fixed in the gastronomic container.

After being removed from their packaging without any additional processing, the files were placed at a length of 19 mm into a static cyclic fatigue testing apparatus and used in the‘Reciproc ALL’ mode of the VDW Gold Reciproc endomotor (VDW, Munich, Germany) until fracture occurred. The time to fracture was determined visually and audibly and recorded using a digital stopwatch with 0.1‐s precision (SEIKO Stopwatch SVAS009, Tokyo, Japan). The lengths of the fractured fragments were measured using a digital caliper (Absolute AOS series 500, Neuss, Germany). Following the completion of the cyclic fatigue test, the fractured fragments were examined under a scanning electron microscope at ×190 magnification to confirm that the fracture occurred as a result of cyclic fatigue, specifically by assessing the presence of crack initiation area and fast fracture zones.

### 2.3 Metallurgical Characterization

To investigate the phase transformation behavior and transformation temperatures of the alloys, differential scanning calorimetry (DSC) analysis was performed using a Pyris Diamond Series instrument (Perkin Elmer, Connecticut, USA) in accordance with the guidelines of the American Society for Testing and Materials (ASTM) (ASTM F2004217 2017). Two unused instruments randomly selected from each group were sectioned into 2–3 mm slices from the tip using a diamond saw. To prevent potential work hardening caused by the cutting process, the terminal 0.5 mm portion of the separated segments was chemically etched for 15 min in a solution consisting of 45% nitric acid, 25% hydrofluoric acid, and 30% distilled water (Wu and Chung 2012). The 20 mg sections were transferred to the differential scanning calorimeter to analyze phase transformations. Measurements were conducted under an argon gas atmosphere within a temperature range of +80 to −80°C, with a heating and cooling rate of 100°C per minute. The transformation temperatures were detected from the intersection of the baseline extrapolation and maximum gradient of the DSC curve. Visual analysis of the transformation temperatures and the charts of the DSC curves was performed using the TA Instruments Universal Analysis program (Waters, New Castle, USA).

Energy dispersive spectroscopy (EDS) analysis was performed on one randomly selected file from each group to evaluate the chemical composition. Files were subjected to a 2‐min acetone bath before analysis. The EDS spectrometer (INCA X‐act 51‐ADD0053, Oxford Instruments, Abingdon, United Kingdom) connected to the SEM was operated at an accelerating voltage of 15.0 kV, a filament current of 3.1 A, and a working distance of 35 mm, under ×75 magnification (Rodrigues et al. 2018). Quantitative analysis was performed using Aztec One Version 3.3 (Oxford Instruments, Abingdon, UK).

### 2.4 Statistical Analysis

Normality of data was assessed using the Shapiro–Wilk test. Data found to be non‐normally distributed were analyzed using the Kruskal‐Wallis test, with post hoc analysis using the Dunn‐Bonferroni test. Results were summarized using mean, standard deviation, median, and interquartile range at a 5% significance level (SPSSv22.0, SPSS Inc., Chicago, USA).

## 3 Results

### 3.1 Design

**FIGURE 4:**
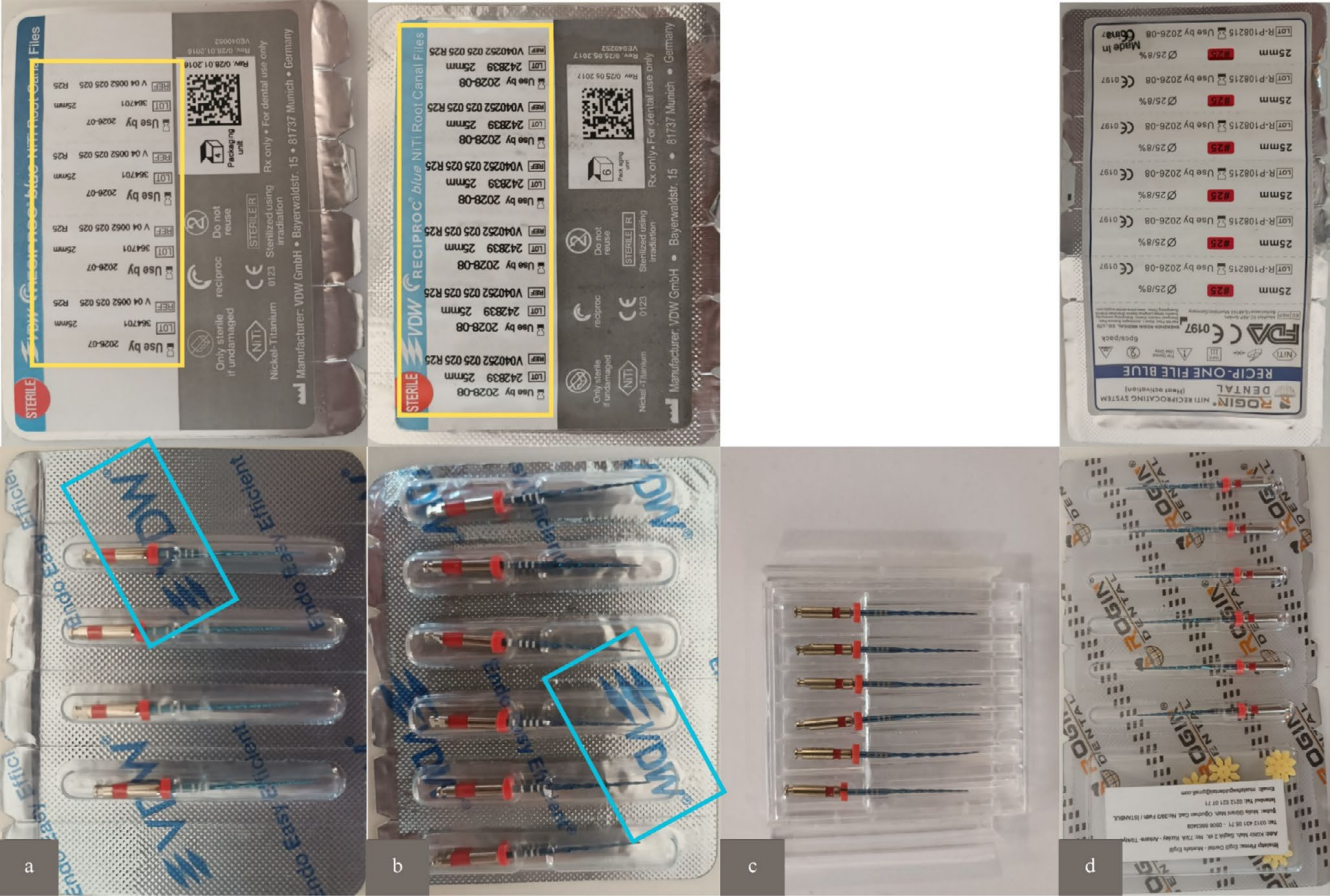
Packaging appearances of the systems (a: Of, b: Cf1, c: Cf2, and d: Rf). The blue and yellow areas highlight the differences between counterfeit and original systems.

The packaging of the Of and Cf2 systems exhibited notable differences. The Cf2 system was delivered to us in a transparent plastic box without any packaging; whereas the Cf1 system's packaging closely resembled that of the original files. In the Cf1 system, the inverted printing of the VDW logo and the use of a bold font for the text on the back were visual details that differed from the original file packaging (Figure 4).

**FIGURE 5:**
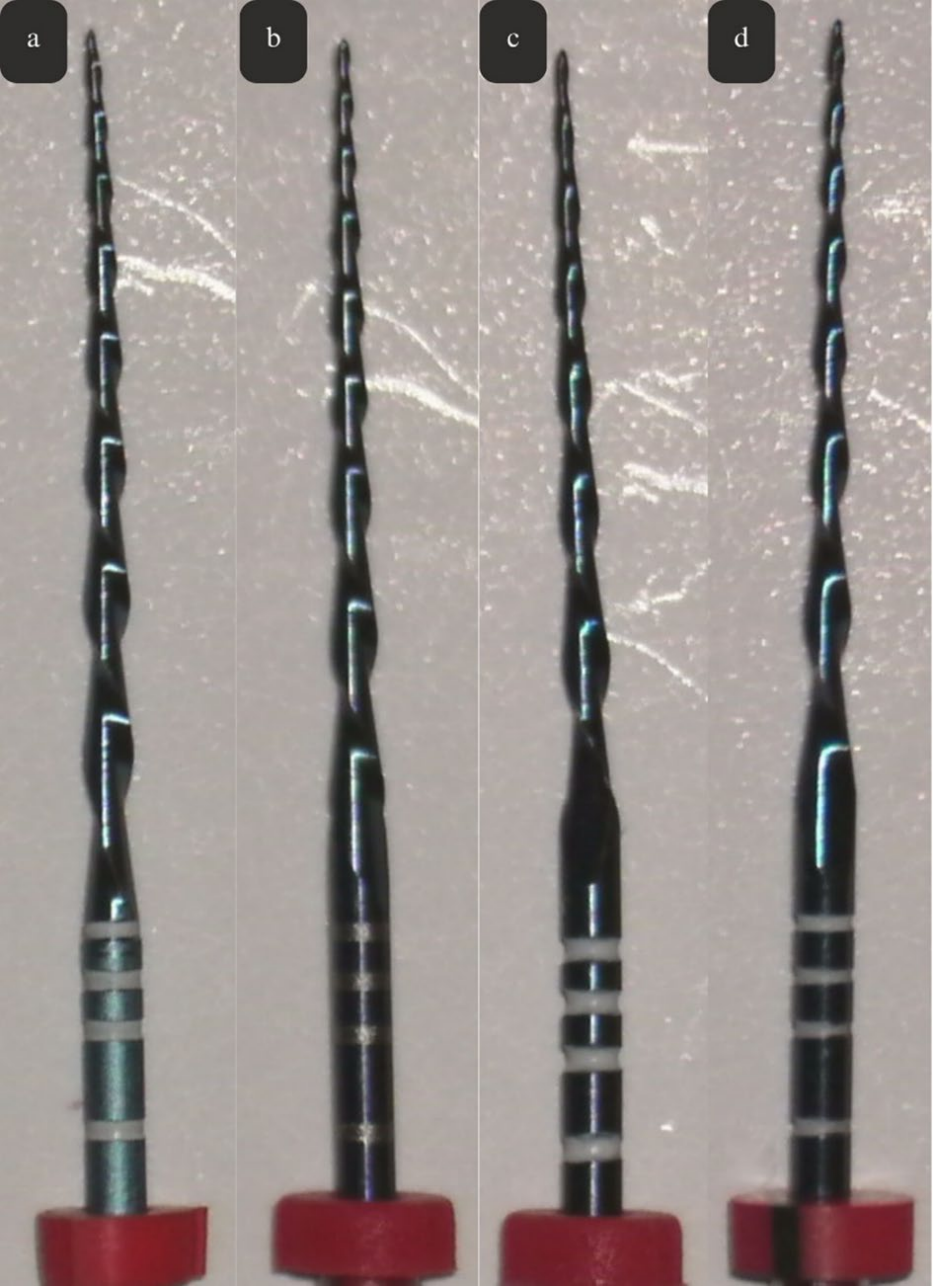
Digital photographs of the systems under a stereomicroscope at ×10 magnification (a: Of, b: Rf, c: Cf1, and d: Cf2).

**FIGURE 6:**
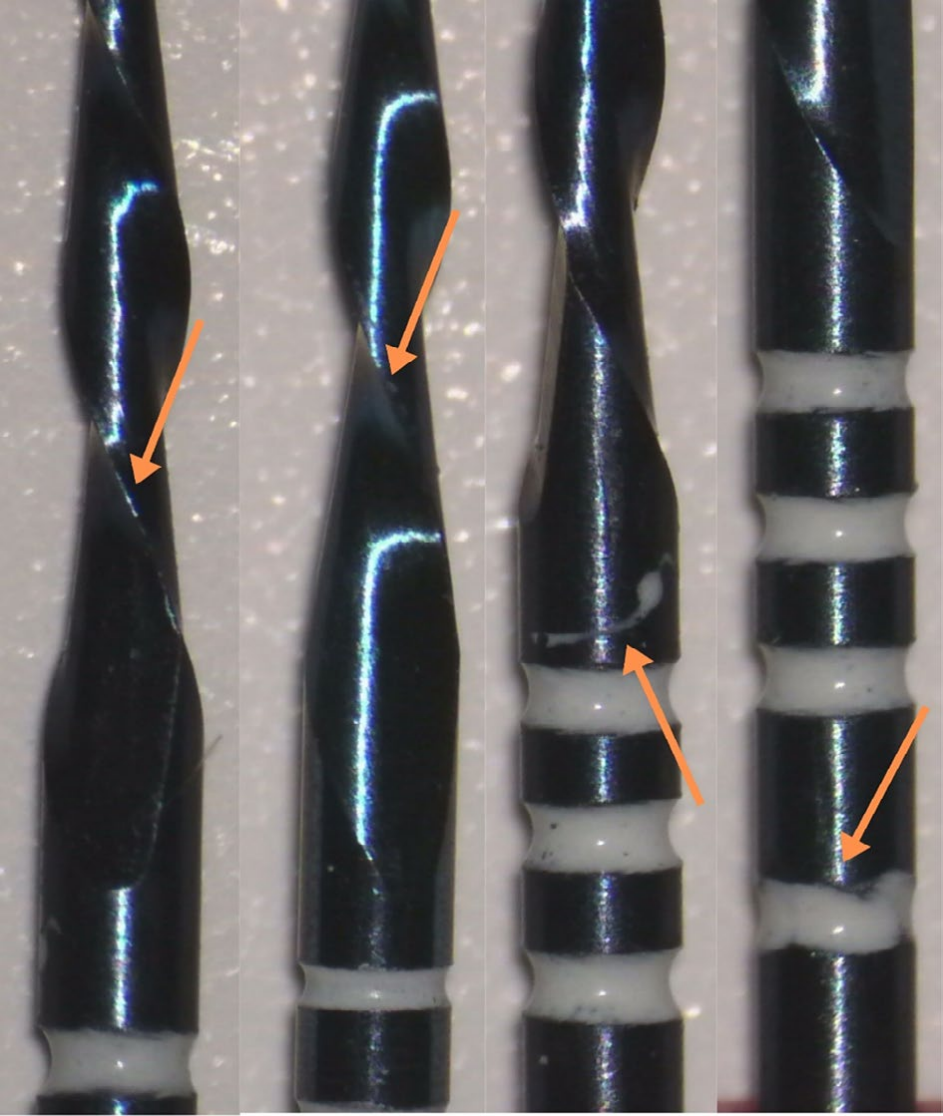
Examination of the cutting regions and measurement lines of counterfeit systems under a stereomicroscope at ×25 magnification. Orange arrows indicate irregularities in the measurement lines and the rough structures on the cutting regions.

At ×10 magnification with a stereomicroscope, no manufacturing defects were observed among the systems. However, the cutting blades of the replica‐like and counterfeit files exhibited a darker blue tone in comparison to the original system. The Cf1 and Cf2 stoppers, which were pale pink in color and did not conform to ISO color coding, differed from the bright red stoppers observed in the Of group. Additionally, black markings were noted on the Cf2 group stoppers (Figure 5). At ×25 magnification under the stereomicroscope, the differences between the systems were more distinctly observed, with manufacturing defects identified in the counterfeit file systems. Additionally, irregular and poorly defined measurement markings were noted in the counterfeit systems, along with evidence of scraping and irregularities on the surface of the cutting regions (Figure 6).

**FIGURE 7:**
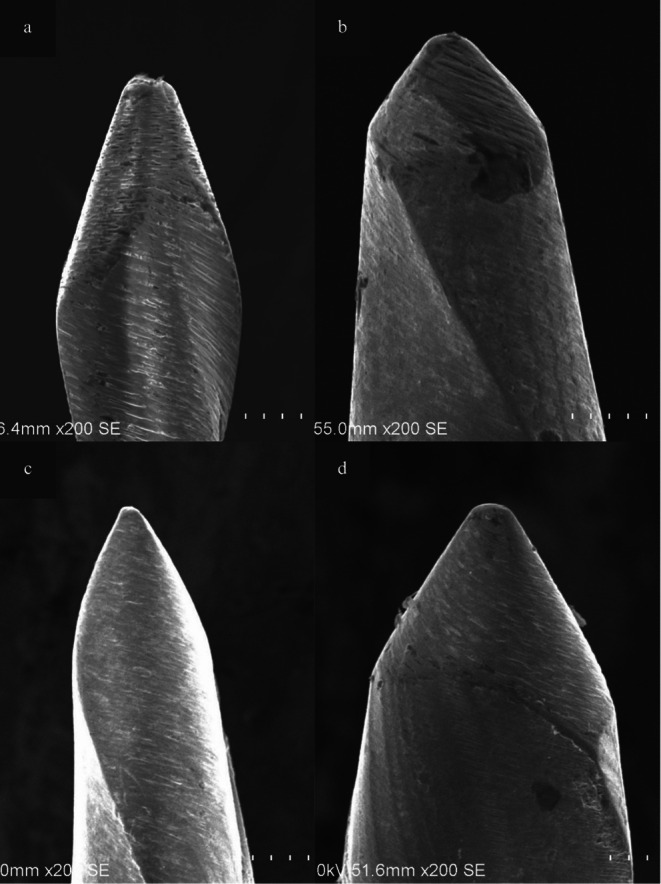
Evaluation of the tip regions under SEM at ×200 magnification (a: Of, b: Rf, c: Cf1, and d: Cf2).

The images obtained at ×50 magnification with the SEM device were analyzed using the Image J software, and the D0 diameter and taper degrees of the systems are presented in Table 1. For the taper parameter, statistically significant differences were found in pairwise comparisons of Cf1‐Cf2, Cf1‐Of, and Cf1‐Rf, with the Cf1 system exhibiting lower taper values compared to the other three systems (*p* < 0.05). For the D0 diameter, statistically significant differences were observed in the pairwise comparisons of Cf1‐Of and Cf1‐Rf, with the Cf1 system exhibiting lower D0 diameter values in comparison to the original and replica‐like file groups (*p* < 0.05). The images obtained at 200× magnification with the SEM device showed that the Of and Rf systems exhibited a non‐cutting tip design, whereas the Cf1 and Cf2 systems demonstrated a cutting tip design (Figure 7).

**TABLE 1:** Evaluation of taper and tip diameter.

sSystems	*n*		Taper	Tip diameter (in mm)
Of	10	Mean + (SD) Median + (IQR) Min–Max	0.08 (±0.002) 0.08 (0.078–0.082)^a^ 0.076–0.083	0.246 (±0.004) 0.247 (0.241–0.249)^d^ 0.239–0.251
Rf	10	Mean + (SD) Median + (IQR) Min–Max	0.082 (±0.005) 0.082 (0.077–0.086)^a^ 0.076–0.091	0.246 (±0.015) 0.252 (0.231–0.258)^d^ 0.22–0.264
Cf 1	10	Mean + (SD) Median + (IQR) Min–Max	0.054 (±0.006) 0.053 (0.047–0.061)^b^ 0.046–0.064	0.181 (±0.02) 0.180 (0.168–0.196)^c^ 0.142–0.215
Cf 2	10	Mean + (SD) Median + (IQR) Min–Max	0.076 (±0.006) 0.075 (0.071–0.082)^a^ 0.067–0.088	0.216 (±0.021) 0.212 (0.199–0.236)^cd^ 0.185–0.248

*Note*: Different superscript letters indicate a statistically significant difference between groups (*p* < 0.005).

Abbreviations: IQR, interquartile range; SD, standard deviation.

The data regarding the precision of the 18–20 mm measurement markings are presented in Table 2. The counterfeit file systems demonstrated measurement marking values deviating from the 18 mm reference distance compared to the original and replica‐like file systems. Specifically, the Cf1 system exhibited values above 18 mm, while the Cf2 system displayed values below 18 mm. Additionally, the Cf2 system showed significant variability, with deviations exceeding 0.1 mm. Similarly, in the evaluation of the 20 mm reference, the counterfeit file systems showed measurement values deviating from the reference compared to the original and replica‐like file systems. Both Cf1 and Cf2 systems demonstrated variability exceeding 0.1 mm, indicating significant deviations.

**TABLE 2:** Evaluation of the precision of 18–20 mm measurement lines.

Systems	*n*		18 mm Marking line (in mm)	20 mm Marking line (in mm)
Of	20	Mean + (SD) Median + (IQR) Min–Max	17.921 (±0.062) 17.93 (17.87–17.96) 17.82–18.05	19.917 (±0.638) 19.92 (19.87–19.95) 19.82–20.1
Rf	20	Mean + (SD) Median + (IQR) Min–Max	17.983 (±0.089) 17.97 (17.912–18.037) 17.87–18.17	20.064 (±0.12) 20.04 (19.982–20.112) 19.94–20.38
Cf 1	20	Mean + (SD) Median + (IQR) Min–Max	18.081 (±0.140) 18.05 (17.985–18.147) 17.88–18.47	20.222 (±0.108)* 20.225 (20.162–20.305) 19.98–20.48
Cf 2	20	Mean + (SD) Median + (IQR) Min–Max	17.779 (±0.145) 17.82 (17.65–17.907)^a^ 17.52–17.96	19.849 (±0.206)* 19.83 (19.725–19.955) 19.48–20.32

Abbreviations: IQR, interquartile range; SD, standard deviation.

^a^ Significant differences in the measurement line position (> 0.1 mm).

### 3.2 Cyclic Fatigue Test

**FIGURE 8:**
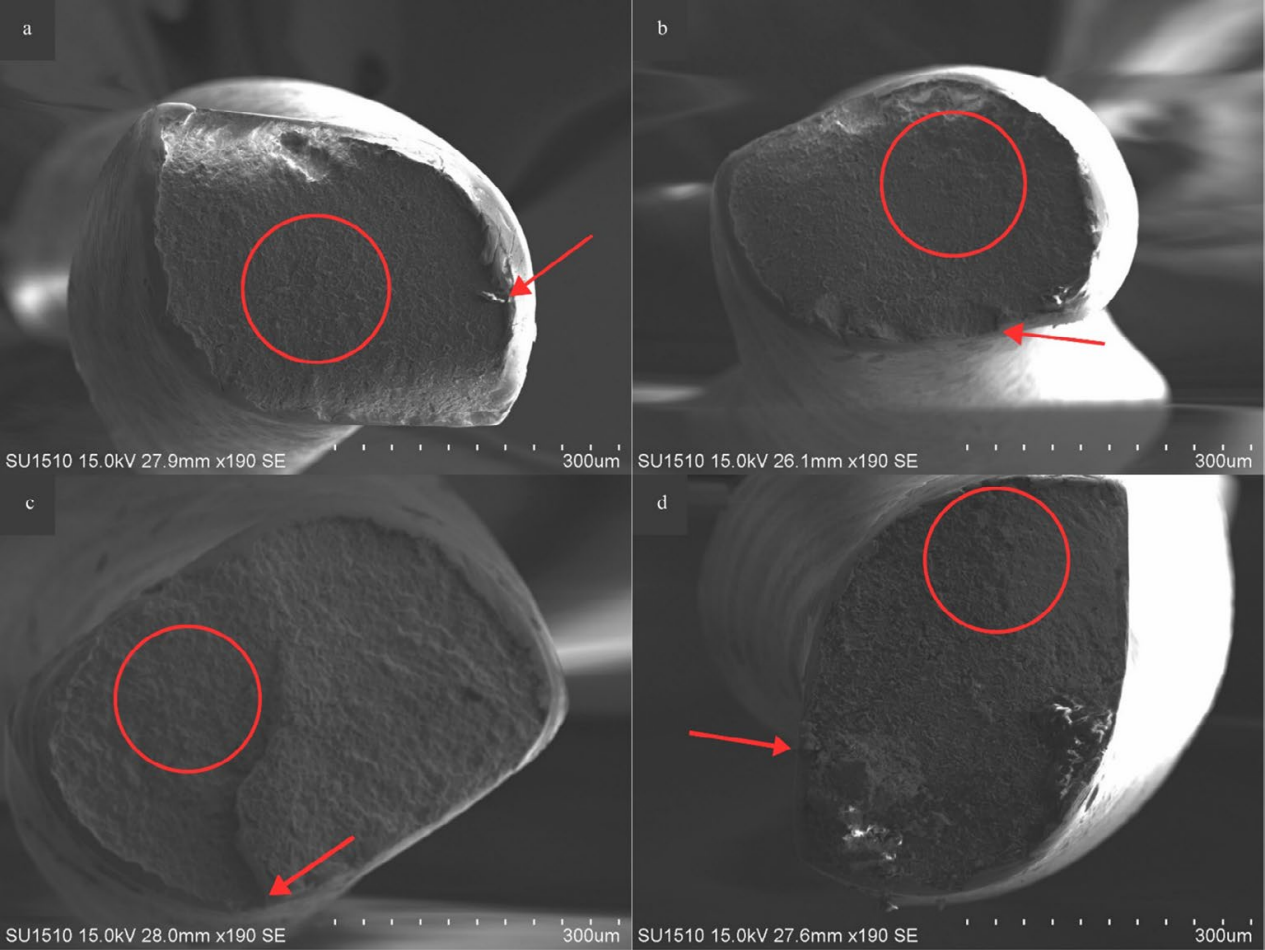
Evaluation of fracture surfaces under SEM at ×190 magnification. Red arrows indicate crack initiation area, and red circles indicate rapid fracture regions. (a: Of, b: Rf, c: Cf1, and d: Cf2).

Time to fracture and lengths of fractured instruments are shown in Table 3. No statistically significant difference was observed between the systems for time to fracture (*p* > 0.05). However, in the pairwise comparisons of fractured fragment lengths, statistically significant differences were noted for the Rf‐Cf2, Rf‐Of, and Rf‐Cf1 comparisons, with the Rf system showing shorter fractured fragment lengths compared to the other systems (*p* < 0.05). At the complete static cyclic fatigue test, the fractured instruments were imaged under the SEM at ×190 magnification. The typical features of cyclic fatigue‐related fractures, including crack initiation area and fast fracture zones, were observed on the fractured surfaces of all groups (Figure 8).

**TABLE 3:** Time to fracture and fragment length values.

Systems	*n*		Time to fracture (in seconds)	Fragment length (in mm)
Of	20	Mean + (SD) Median + (IQR) Min–Max	257.7 (±18.1) 258.9 (246.6–270)^a^ 223–291.1	3.47 (±0.2) 3.38 (3.32–3.56)^b^ 3.24–3.96
Rf	20	Mean + (SD) Median + (IQR) Min–Max	253.1 (±58.2) 254.2 (194.8–311.3)^a^ 170–343.5	2.71 (±0.22) 2.73 (2.5–2.9)^c^ 2.32–3.07
Cf 1	20	Mean + (SD) Median + (IQR) Min–Max	304 (±135.8) 252.1 (204.1–432.5)^a^ 102.3–618.3	4.18 (±0.98) 4.42 (3.47–5.02)^b^ 1.68–5.54
Cf 2	20	Mean + (SD) Median + (IQR) Min–Max	254.6 (±133) 256.7 (142–370.3)^a^ 69.2–545.1	3.53 (±1.07) 3.49 (2.36–4.31)^b^ 1.88–5.27

*Note*: Different superscript letters indicate a statistically significant difference between groups (*p* < 0.005).

Abbreviations: IQR, interquartile range; SD, standard deviation.

**FIGURE 9:**
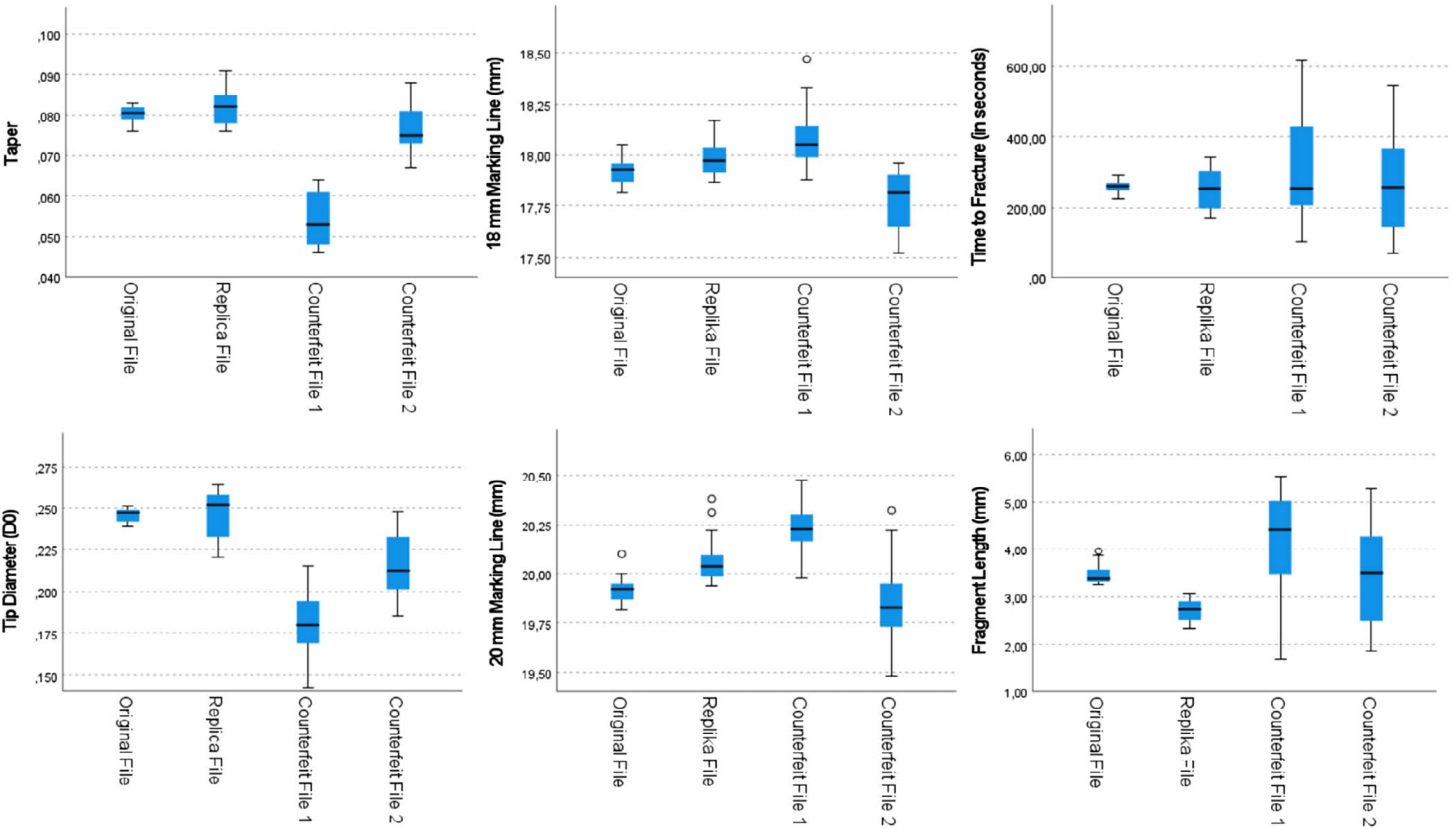
Box‐plot graphs illustrating all examined parameters.

Box plot graphs illustrating the evaluation of taper, D0 diameter, measurement line precision, time to fracture, and fractured fragment length are presented in Figure 9. The data for counterfeit file systems exhibited greater variability compared to the other groups.

### 3.3 Metallurgical Characterization

Table 4 shows the Af (austenitic finish temperature) values and atomic level nickel‐titanium (Ni‐Ti) compositions of the instruments examined. The Ni‐Ti levels were found to be similar between the systems. According to the DSC results, the highest Af value was observed in the Rf system, and the lowest in the Of system. The Af temperatures of the Rf, Cf1, and Cf2 systems exceeded the experimental temperature of 37°C, indicating a martensitic crystallographic structure at body temperature for these three systems. In contrast, the Af value of the Of system (33.97°C) was below body temperature, indicating an austenitic crystallographic structure.

**TABLE 4:** Ni–Ti compositions and Af values.

Systems	Nickel–titanium (atomic %)	Af (°C)
Of	50.78–49.22	33.97
Rf	50.41–49.59	52.83
Cf 1	50.66–49.34	45.50
Cf 2	50.44–49.56	41.84

## 4 Discussion

Studies comparing the packaging appearances of original and counterfeit systems have reported that counterfeit systems are packaged similarly to the original systems (Rodrigues et al. 2018; Madytianos et al. 2023). Rodrigues et al. (Rodrigues et al. 2018) noted that the packaging of counterfeit Reciproc files closely resembled that of the original system and observed that the stopper colors of counterfeit systems were pink compared to the original systems. Madytianos et al. (Madytianos et al. 2023) observed that in counterfeit Mtwo packaging, the placement of the word “sterile” was different, and in counterfeit Protaper Next packaging, the font differed from that of the original system. Investigators noted these discrepancies upon visual examination of the systems. Similarly, in our study, counterfeit file systems exhibited a paler stopper color compared to the red tones present in the stoppers of the original system. Additionally, the Cf2 system in our study was packaged in a manner entirely different from the original system. In contrast, the Cf1 system was packaged in a way that closely resembled the original system, consistent with previous findings.

To differentiate counterfeit systems from original systems, previous studies have examined systems under SEM at magnifications such as ×271 (Noenko et al. 2023), ×500 (Martins et al. 2022), and ×350 (Madytianos et al. 2023), reporting that surface irregularities and crack lines were more frequently observed in counterfeit systems. In our study, no surface defects were detected under a stereomicroscope at ×10 magnification; however, at ×25 magnification, production defects such as surface roughness were observed in counterfeit systems. These findings suggest that manufacturing defects and surface differences in counterfeit systems can be identified without the need for high magnification, facilitating their differentiation from original systems.

Replica‐like systems have been reported to exhibit tip designs similar to the original system (Martins et al. 2020; dos Reis et al. 2023). In contrast, counterfeit systems have been shown to feature an active tip design, independent of the original system (Rodrigues et al. 2018; Noenko et al. 2023). Consistent with these findings, our study also demonstrated that replica‐like systems exhibited a passive tip design, aligning with the original system, while counterfeit systems exhibited an active tip design. The differences in tip design observed in counterfeit systems are thought to potentially predispose them to complications during clinical use.

Statistical analysis of the taper and tip diameter values showed that the original and replica‐like systems exhibited similar taper and tip diameter values. In contrast, the Cf1 system showed significantly lower taper and tip diameter values than the original system; while the Cf2 system, although not statistically significant, showed lower median values than the original system. Contrary to these findings, a study in the literature reported that counterfeit systems were designed with larger dimensions than the original system (Noenko et al. 2023). Based on these results, it was shown that the counterfeit systems did not meet the claimed dimensional values. Furthermore, considering that increased taper and tip diameter values increase metal mass and consequently production costs, we hypothesize that some counterfeit systems, as observed in our study, were manufactured with reduced dimensions to minimize production costs.

The evaluation of the accuracy of measurement lines was based on the 0.1 mm reference value accepted in previous studies (Martins et al. 2020; Martins et al. 2022; Martins et al. 2021). While the replica‐like systems showed deviations similar to the original system, the counterfeit systems showed deviations exceeding 0.1 mm. The incorrect line levels observed in the counterfeit systems indicate that these systems are unreliable.

Unlike torsional stress‐induced fracture, there are no specific guidelines or international standards for evaluating cyclic fatigue resistance using test setups (Zhou et al. 2013). In a review published by Hülsmann et al. (Hülsmann et al. 2019), it was noted that test setups for cyclic fatigue resistance typically use a 5 mm radius of curvature and a 60° angle of curvature, respectively. To ensure standardization and enable comparison with other studies, we designed the artificial canal in our test setup with a 5 mm radius of curvature and a 60° angle of curvature. Studies regarding the dimensions of artificial canals and their compatibility with the instruments studied are limited. According to ISO standards, a tolerance of 0.02 mm is considered acceptable for endodontic instruments (International Organization for Standardization 2019). To ensure that the instruments could rotate freely and to account for this tolerance, the artificial canal was designed to be 0.02 mm larger than the claimed dimensions of the systems.

The increase in temperature has been reported to reduce the cyclic fatigue resistance of Ni‐Ti files (De Vasconcelos et al. 2016; Dosanjh et al. 2017; Gündoğar et al. 2019), and it has been emphasized that test setups should be performed at body temperature to simulate clinical conditions (Savitha et al. 2022). In this study, temperature elevation and monitoring in the test setup were achieved using a sous vide machine. Previous studies utilized aquarium‐type heaters for temperature regulation and thermometers for monitoring (Keskin et al. 2021; Klymus et al. 2019; Plotino et al. 2018). Considering its integrated components, vertical placement capability, and space‐saving advantage in test setups, the sous vide machine is expected to be widely employed in future studies.

The mean time to fracture for the Reciproc Blue R25 file, selected as the original system, was determined to be 257.7 s in our study. While similar fracture times were observed in previous studies using comparable test setups (Plotino et al. 2018; Keleş et al. 2019; Serafin et al. 2019; Topçuoğlu et al. 2018), fracture occurrences at different durations have also been reported in the literature (Adigüzel and Turgay 2017; Alcalde et al. 2018; Al‐Obaida et al. 2019). The lack of standardization in cyclic fatigue test setups is likely to contribute to the variability in results, with differences in the material composition and dimensions of artificial canals being key factors.

No statistically significant difference was found when comparing the fracture times of the original and replica‐like systems. While similar results have been reported in previous studies (dos Reis et al. 2023; Martins et al. 2021), other studies have shown that original systems have higher cyclic fatigue resistance compared to replica‐like files (Martins et al. 2020; Uslu et al. 2023). The similarity in fracture times between replica‐like files and the original system in our study may be attributed to our DSC results. Consistent with previous studies, the original Reciproc Blue system exhibited the austenite phase at body temperature (Odgerel et al. 2024; Silva et al. 2024). In contrast, the replica‐like Recip One Files system exhibited the martensite phase at body temperature. It has been reported that files in the martensitic phase have higher cyclic fatigue resistance than those in the austenitic phase (Ruiz‐Sánchez et al. 2020; Santoro et al. 2001).

Similarly, no statistically significant difference in fracture times was found between the original and counterfeit systems. This result is completely contrary to the findings of similar studies in the literature (Martins et al. 2022; Rodrigues et al. 2018; Madytianos et al. 2023; Martins et al. 2021; Ertas et al. 2014). The different cross‐sectional design (Ertas et al. 2014), the rough surface structure (Martins et al. 2022; Madytianos et al. 2023), and the presence of the austenite phase in these files according to DSC results (Martins et al. 2022) have been identified as factors contributing to the lower performance of counterfeit systems. In our study, we believe that three parameters contributed to the similar fracture times of the counterfeit systems compared to the original systems. The first parameter is the DSC results. Unlike the original system, the counterfeit systems were found to be in the martensitic form at body temperature. The similar cyclic fatigue resistance observed in these systems to the original system can be explained by the flexibility of the martensitic phase. The effect of taper and D0 diameter values on cyclic fatigue resistance is the second parameter. It has been reported that a reduction in taper and D0 diameter values increases the cyclic fatigue resistance (Rodrigues et al. 2018; Silva et al. 2024). In our study, the Cf1 system showed statistically significant lower values for both taper and D0 diameter compared to the original file system. Although not statistically significant, the Cf2 system showed lower median values for taper and D0 diameter compared to the original system. The effect of artificial canal size on cyclic fatigue resistance is the third parameter. It has been reported that the time to fracture increases with increasing canal size in cyclic fatigue test setups conducted in artificial canals (Bürklein, Maßmann, et al. 2021; Plotino et al. 2010). This phenomenon can be explained by the reduction in file‐to‐canal fit due to the increase in artificial canal size, which resulted in the file having less curvature and being subjected to less stress within the canal. Counterfeit systems that did not meet the claimed taper and size showed a low angle of curvature and a high radius of curvature in the artificial canal due to the tendency of the file to return to its original shape.

Another notable finding for counterfeit systems is the significantly higher standard deviation of the data compared to the other groups. For all parameters evaluated (taper, D0 diameter, 18–20 mm accuracy assessment, fracture times, fragment length), the data for counterfeit systems were more widely distributed compared to the other systems. This result highlights the lack of standardization in counterfeit systems. It is believed that inconsistencies in manufacturing procedures and deficiencies in quality control have contributed to this issue. Although the alternative file systems demonstrated comparable cyclic fatigue resistance to the original system, the null hypothesis was rejected due to significant differences in design and metallurgical properties.

In terms of fragment length, the replica‐like system showed significantly lower fragment length compared to both the original and counterfeit systems. Considering the similarities in size and chemical composition, it is believed that a potential difference in the material mass of the replica‐like system may have contributed to this result. It has been reported that differences in material mass can influence the fragment length (Bürklein, Zupanc, et al. 2021). According to the SEM‐EDS results, the Ni‐Ti levels were similar among the systems. Consistent with other studies (Martins et al. 2020; Madytianos et al. 2023; Martins et al. 2021), this finding indicates that the material composition did not affect the cyclic fatigue resistance of the systems examined in our study.

The comparison of the design, mechanical, and metallurgical properties of original, replica‐like, and counterfeit files operating in reciprocating motion within a single study, as well as the interpretation of how dimensional and design discrepancies in counterfeit systems influence cyclic fatigue resistance tests, are considered to be among the strengths of our study. The selection of systems from different production lots would provide more comprehensive results, and this constitutes a limitation of the study. The failure of counterfeit systems to meet the claimed dimensions and tapers indicates that future studies will require multiple artificial canals specific to original and counterfeit systems. Additionally, performing further tests such as cutting efficiency, shaping ability, and torsional resistance would offer more comprehensive insights into the mechanical properties of both replica‐like and original instruments.

## 5 Conclusions

- Replica‐like systems demonstrated comparable design, mechanical, and metallurgical properties to the original file systems, with the exception of the DSC findings.
- Counterfeit file systems were distinguished from the original system by the presence of manufacturing defects observed under a stereomicroscope. Due to their inconsistency in standardization and design, the use of these systems is considered less reliable and may predispose clinicians to potential clinical complications.

## Author Contributions

**Mert Unal:** conceptualization, investigation, writing – original draft, methodology. **Elif Bahar Cakici:** software.

## Conflicts of Interest

The authors declare no conflicts of interest.

## Data Availability

The data that support the findings of this study are available from the corresponding author upon reasonable request.
